# The Interplay of Chronic Hepatitis C and COVID-19: Implications for Prognosis and Treatment

**DOI:** 10.7759/cureus.66639

**Published:** 2024-08-11

**Authors:** Stefan D Lazar, Andreea F Stoenescu, Corneliu Petru Popescu, Simin Florescu, Geta Vancea, Petre Calistru

**Affiliations:** 1 Infectious Diseases and Tropical Medicine, Dr Victor Babes Hospital of Infectious and Tropical Diseases, Carol Davila University of Medicine and Pharmacy, Bucharest, ROU; 2 Infectious Diseases, Carol Davila University of Medicine and Pharmacy, Bucharest, ROU

**Keywords:** mortality, severe disease, sars-cov2, covid-19, chronic hepatitis c (chc)

## Abstract

Introduction

Chronic hepatitis C (CHC) remains a significant public health concern due to both hepatic and extrahepatic manifestations associated with substantial morbidity and mortality. The emergence of SARS-CoV-2 has raised concerns about the outcomes of COVID-19 in CHC patients.

Method

We conducted a retrospective analysis of patients with CHC and SARS-CoV-2 infection admitted to a tertiary care hospital between 2020 and 2023. We performed a global analysis of the entire batch of patients and, later, we evaluated the patients according to the severity of the SARS-CoV-2 infection

Results

The cohort included 89 patients (63 females, 26 males) with a median age of 65 years. Most patients were hospitalized in 2021. Common clinical manifestations included fever, cough, digestive symptoms, and headache. The most frequent comorbidities were renal disease, thyroid disorders, and cancer. Univariate logistic regression analysis identified older age, hospitalization in 2021, and respiratory failure as risk factors for severe COVID-19. Elevated lactate dehydrogenase levels were also associated with an increased risk of severe COVID-19. Regarding CHC, detectable hepatitis C virus viremia was associated with more severe liver disease (p<0.01).

Conclusion

Patients with CHC and SARS-CoV-2 infection have a substantial risk of severe outcomes. Early identification and management of these patients are crucial to improve their prognosis.

## Introduction

The emergence of the novel coronavirus disease 2019 (COVID-19) has cast a long shadow over global health, disrupting lives and claiming millions worldwide [1]. While the majority of cases present with mild to moderate symptoms, a significant proportion of individuals, particularly those with underlying comorbidities, face severe complications, including respiratory failure and multi-organ dysfunction [2,3]. Among these vulnerable populations, hepatitis C virus (HCV) infection has emerged as a potential risk factor, raising concerns about its impact on COVID-19 outcomes [4,5].

HCV, a bloodborne virus, primarily targets the liver, leading to chronic inflammation and, in some cases, cirrhosis and liver cancer [6,7]. The global prevalence of HCV infection is estimated at around 50 million, with a substantial burden in low- and middle-income countries [8]. Despite the revolutionary advancements in antiviral therapies for HCV, a substantial proportion of infected individuals remain undiagnosed or untreated, perpetuating the risk of HCV-related complications [9].

Numerous studies have delved into the association between HCV infection and COVID-19 outcomes, revealing a consistent pattern of increased risk for severe COVID-19, hospitalization, and mortality among HCV-infected individuals [10-12]

This study delves into the potential association between the clinical presentation, laboratory findings, and outcomes of patients hospitalized in our department with documented HCV infection and COVID-19. Specifically, we aim to investigate the course of SARS-CoV-2 infection in these patients, including the progression of symptoms, and the severity of the illness. Furthermore, we will explore the relationship between the severity of COVID-19 infection and the degree of liver function impairment in this patient population. By analyzing these factors, we hope to gain valuable insights into the potential impact of HCV on COVID-19 outcomes and identify any specific considerations for managing patients with both conditions.

## Materials and methods

Study design and patient selection

We conducted a single-center, retrospective cohort study design. The study included all patients over 18 years of age with known chronic HCV infection and COVID-19 who were hospitalized between January 2020 and December 2023 at the ”Dr. Victor Babeș” Clinical Hospital of Infectious and Tropical Diseases in Bucharest, Romania.

Inclusion criteria

Adult patients (18 years old or more) diagnosed with SARS-CoV-2 infection and who had anti-HCV antibodies present at admission.

Exclusion criteria

Exclusion criteria consisted of viral co-infections (patients with other viral hepatitis or HIV co-infections), individuals under the age of 18, and pregnant or breastfeeding women.

Patient categories

Patients were divided into several subgroups based on their HCV treatment status. Those with a treatment history of interferon or direct-acting antivirals (DAAs) and obtained, as a result, sustained virologic response (SVR), those who were untreated (with detectable ARN-HCV), and those with cirrhosis (treated or untreated before).

Data collection

Demographic data (age, sex, comorbidities), clinical information, and laboratory findings were collected from the patient's medical records. Laboratory tests included white blood cell counts, platelet counts, inflammatory markers, liver function tests (alanine aminotransferase, aspartate aminotransferase, bilirubin, lactate dehydrogenase), and prothrombin time. The diagnosis of COVID-19 was confirmed by detecting viral RNA from nasopharyngeal secretions using either RT-PCR or antigen testing. SARS-CoV-2 infection was classified into three categories based on severity: mild, moderate, and severe. COVID-19 severity was categorized based on respiratory symptoms. Mild cases presented with a low-grade fever and no signs of pneumonia. Moderate cases included fever and signs of non-severe pneumonia, without the need for oxygen support. Severe cases were defined by respiratory failure requiring mechanical ventilation, rapid breathing (respiratory rate exceeding 30 breaths per minute), and low oxygen saturation (SpO2 below 94% measured by pulse oximetry).

We performed a global analysis of the entire batch of patients and, later, we evaluated the patients according to the severity of the SARS-CoV-2 infection. Finally, we studied the influence of HCV infection on the evolution of COVID-19. In this sense, we accumulated the risk factors of severe evolution for COVID-19 known in the literature (obesity, hypertension, diabetes) [13-15], to which we assigned one point each and integrated them into a risk factor score ( RF score) to which we assigned one point to each element so that this score was from 0 to three points.

Data analysis

For assessing predictors' importance in the outcome of interest including HCV status (deceased or survivor) or in the severity of COVID (severe versus non-severe) we employed the binomial logistic regression with link logit function.

For statistical analysis, R, program version 4.4.0 [Copyright (C) 2024 The R Foundation for Statistical Computing, R Core Team (2024). A: A language and environment for statistical computing. R Foundation for Statistical Computing, Vienna, Austria.]

For all statistical tests, the cut-off α level was 0.05, and all p-values below 0.05 were considered significant.

Ethical considerations

The study was approved by the Ethics Committee of the Dr. Victor Babeș Clinical Hospital of Infectious Diseases and Tropical Diseases number 19901 on 28th December 2020. All patient data was handled confidentially in accordance with ethical guidelines.

## Results

The analyzed cohort included 89 patients aged between 34 and 92 years. Most of the patients were admitted in 2021. Among the clinical manifestations, fever, and cough were most frequently present, followed by digestive manifestations and headache. Most frequently, patients had associated thyroid and oncological pathologies.

The overall analysis of patient groups can be found in Table 1. We assessed the importance of demographic and clinical factors (signs and symptoms, comorbidities) in the patient’s outcome.

**Table 1:** Univariate logistic regression to identify predictors significance in dead outcome BMI: body mass index, RF: risk factor.

Predictor	N	Exitus (N)	OR (95% CI)1	P -value
Gender				
Female	63	5	-	
Male	26	5	2.76 (0.70 to 10.9)	0.136
Age	89	10	1.04 (0.98 to 1.11)	0.189
Area				
Rural	18	3	-	
Urban	71	7	0.55 (0.13 to 2.76)	0.419
IMC/ BMI	50	5	1.09 (0.95 to 1.25)	0.193
Fever				
Yes	49	6	-	
No	40	4	0.80 (0.19 to 3.00)	0.739
Cough				
Yes	48	5	-	
No	41	5	1.19 (0.31 to 4.61)	0.791
Cephalea				
Yes	23	3	-	
No	66	7	0.79 (0.20 to 3.94)	0.750
Anosmia				
Yes	12	1	-	
No	77	9	1.46 (0.24 to 28.2)	0.733
Ageusya				
Yes	9	1	-	
No	80	9	1.01 (0.16 to 19.9)	0.990
Digestive manifestations				
Yes	26	1	-	
No	63	9	4.17 (0.72 to 79.0)	0.187
Vomiting				
Yes	9	1	-	
No	80	9	1.01 (0.16 to 19.9)	0.990
Oxygen Saturation	89	10	0.86 (0.78 to 0.94)	0.002
RF Score	89	10	1.35(0.69-2.64)	0.377
Yes	22	4	-	
No	67	6	0.44 (0.11 to 1.89)	0.244
Psychiatric affections				
Yes	8	1	-	
No	81	9	0.88 (0.13 to 17.3)	0.906
Kidney diseases				
Yes	13	3	-	
No	76	7	0.34 (0.08 to 1.76)	0.158
Oncological diseases				
Yes	10	3	-	
No	79	7	0.23 (0.05 to 1.23)	0.062
Thyroid conditions				
Yes	14	3	-	
No	75	7	0.38 (0.09 to 1.95)	0.201
Antiviral treatment				
Yes	40	4	-	
No	49	6	1.26 (0.33 to 5.23)	0.739

From the univariate analysis summary (Table 1), we see that O2 saturation is a significant protective factor [odds ratio (OR) =0.86, 95% confidence interval (CI) (0.78,0.94), p=0.002)]. An increase of only 1% in O2 saturation leads to a decrease of up to 14% in the probability of a fatal outcome. Also, we could consider oncological comorbidities as being a risk factor to be considered even though has a marginal significant value [OR =0.23, 95% CI (0.05, 1.23), p=0.062], leading to a significant increase of approximative four times in odds of fatal outcome. Also, the administration of the antiviral treatment had no statistical significance in the evolution of the patients.

From evaluating the paraclinical variables, we retained only a few with statistical significance, which we present below. Related to them, thrombocytopenia is a significant risk factor [OR =0.18, 95% CI (0.04,0.70), p=0.019] patients without it presented a risk of death more than five (5.56) times lower than patients with it. Ferritin was also a significant risk factor associated with an approximately 18% increase in the risk of death and a 100-unit increase in lactate dehydrogenase (LDH) levels is associated with a 145% (almost five times) increase in the risk of death. For patients without cirrhosis, the risk of death was nearly four times lower but with marginal significance [OR=0.23, 95%CI(0.05, 1.23)] (Appendix 1).

Regarding paraclinical investigations, we can say that for patients who presented changes on the CT scan, the risk of death was five times higher. CT findings showed the presence of ground-glass opacities in both lung fields (Appendix 1).

In Table 2 we assessed the importance of demographic and clinical factors (signs and symptoms, comorbidities) in the patient’s COVID-19 severity. We present in the table below only the data with relevant statistical significance.

**Table 2:** Univariate logistic regression to demographic and clinical variables significance in having severe form of COVID-19 RF: risk factor.

Predictor	N	Severe Form (N)	OR (95% CI)	p-value
Age	89	41	1.04 (1.01 to 1.08)	0.036
Year (2021)	43	27	3.23 (1.29 to 8.44)	0.014
Oxygen Saturation	89	41	0.58 (0.45 to 0.71)	<0.001
RF Score	89	27	1.72(1.06-2.79)	0.027
Absence of thyroid conditions	75	31	0.28 (0.07 to 0.93)	0.047

From Table 2 we can see that: older patients had higher odds of severe forms, an increase of one year being associated with a 4% increase in the odds of severe forms [OR=1.04, 95% CI (1.01,1.08), p=0.036].

A significant risk predictor was in the year 2021 compared to patients from 2020, patients from 2021 had 3.2 times higher odds of severe forms [OR=3.23, 95% CI (1.29,8.44), p=0.014].

Patients who had a risk factor (RF) score higher were 72% more likely to present a severe form of COVID-19 than patients with lower values of the score.

Patients who did not have thyroid diseases had odds of severe forms almost four times lower, compared to patients with thyroid diseases.

Among the possible predictors studied, we found that gender, and clinical manifestations (fever, cough, headache, digestive manifestations) had no statistical significance regarding severe versus non-severe forms.

Regarding treatment, we conducted a simple descriptive analysis. Hepatitis C viremia was a predictive factor for unfavorable outcomes in those who received corticosteroid therapy: nine patients had severe forms, and 15 had non-severe forms with undetectable viremia. Among patients with detectable viremia, the majority developed a severe form of COVID-19 (30/32).

In Table 3 we summarize the influence of paraclinical investigations on COVID-19 severity.

**Table 3:** Univariate logistic regression to the significance of preclinical predictors in having a severe form of COVID-19 ALT: alanine aminotransferase, AST: aspartate aminotransferase, LDH: lactate dehydrogenase.

Predictor	N	Severe Form	OR (95% CI)1	P-value
Leukopenia				
Yes	31	13		
No	58	28	1.29 (0.54 to 3.16)	0.568
Inflammatory syndrome				
Yes	79	39		
No	10	2	0.26 (0.04 to 1.10)	0.098
Thrombocytopenia				
Yes	30	16		
No	58	24	0.62 (0.25 to 1.50)	0.287
Prothrombin Index <70 (%)	81	36	0.99 (0.96 to 1.01)	0.282
Ferritin/100	51	31	1.06 (0.96 to 1.17)	0.262
Interleukin 6 (pg/ml)	19	9	1.00 (1.00 to 1.02)	0.256
ALT on admission (U/L)	65	27	0.98 (0.94 to 1.02)	0.430
ALT during hospitalization (U/L)	39	13	0.99 (0.94 to 1.03)	0.606
AST on admission (U/L)	34	12	0.94 (0.82 to 1.07)	0.337
AST during hospitalization (U/L)	24	10	1.00 (0.89 to 1.13)	0.959
LDH / 100	77	36	3.42 (1.99 to 6.74)	<0.001
CT findings				
Yes	17	14		
No	72	27	0.13 (0.03 to 0.44)	0.003
Cirrhosis				
Yes	10	9		
No	79	32	0.08 (0.00 to 0.43)	0.017

Regarding paraclinical investigation from Table 3, we can say that patients without inflammatory syndrome had four times lower odds of severe forms, compared to patients with it.

LDH value proved to be a robust negative predictor, an increase in 100 units of LDH value being associated with odds almost 3.5 higher of having a severe form of COVID-19.

Abnormal thoracic computed tomography results are a strong risk factor also, people who do not have these abnormalities have almost eight times lower risk [OR= 0.13, 95% CI (0.03 to 0.44), p=0.003], and patients without cirrhosis had more than 10 times lower odds of severe forms of the disease, compared to patients with cirrhosis [OR= 0.08, 95% CI (0.00 to 0.43), p=0.017].

The HCV infection status was known in 63 non-cirrhotic patients from the studied group, 26 of them having detected viremia. We compared these patients with the rest (37) whom we considered the control subgroup (Table 4).

**Table 4:** Impact of Hepatitis C (HCV) on COVID-19, outcome RF: risk factor, LDH - lactate dehydrogenase, ALT - alanine aminotransferase.

	Undetected viremia, n=37	Detected viremia, n=26 (without cirrhotics)	p
Age (mean)	62.81 (58.63-6699)	66.96 (61.76-72.17)	0.206
Gender			0.305
Male	13(35.1%)	6(23.1%)	
Female	24(64.9%)	20(76.9%)	
Clinical form of COVID-19			<0.001
Severe	28(75.7%)	32(88.9%)	
Moderate/Mild	9(87.5%)	4(11.1%)	
RF score (mean)	0.81 (0.51-1.12)	0.96 (0.65-1.27)	0.503
Number of hospitalization days (mean)	13.46 (10.5-16.42)	14.69 (11.95-17.43)	0.213
Pulmonary changes			0.339
No change or interstitial accentuation	9(25.%)	4(15%)	
Infiltrates or gound-glass	26(75%)	22(85%)	
Laboratory findings			
LDH (U/L) (mean)	284.5 (244.2-324.7)	381.9 (291.5-472.2)	0.033
ALT (U/L) (mean)	33.28 (26.43-40.13)	35.71 (28.9-42.5))	0.623
Platelets (mean) (valx10^9^)	188 (157.4-219.9)	229 (187.5-271.0)	0.110
Ferritin (ng/ml) (mean)	586.61 (308.0-865-12)	952.17 (609.4-1294-8))	0.052
Leukopenia (yes, %)	14(37.8%)	6(26.1%)	0.348
Treatment			
Corticosteroid (yes)	24(64.9%)	23(88.5%)	0.034
Immunomodulators (yes)	8(21.6%)	14(53.8%)	0.008
Outcome (n, %)			0.086
Favorable	35(94.6%)	21(80.8%)	
Unfavorably (deceased)	2(5.4%)	5(19.2%)	

From the table above, we found that more severe forms of the disease were registered in patients who presented detectable HCV viremia (p<0.01). Also for this category of patients, LDH and ferritin were significant severity factors. RF score did not have a significant influence on the two patient subgroups. Regarding the treatment, due to the severity of the disease, patients with detectable HCV viremia required corticotherapy or immunomodulatory treatment in a significantly higher number than in the case of the other subgroup.

Although patients with cirrhosis represented a small number, we attempted to differentiate them based on the presence of HCV viremia (Appendix 2).

We did not find any statistically significant differences, and the presence of viremia in patients with cirrhosis did not seem to influence their outcome.

## Discussion

The impact of co-infection with chronic hepatitis C (HCV) and COVID-19 is not yet fully understood. While some studies have explored this area, they often involve limited numbers of patients. Additionally, existing research might include data from individuals with various chronic liver conditions besides HCV, making it difficult to isolate the specific effects of HCV in this context.

It is known that as the COVID-19 pandemic evolved, there were variations in the severity of the disease. For the patients in the studied group, the most severe or unfavorable developments were recorded in 2021.

Prior studies have evaluated routine blood parameters for liver function assessment and investigated its alterations in COVID-19 patients. Some of their observations aligned with our findings, regarding significant elevations in aspartate aminotransferase (AST), alanine aminotransferase (ALT), and LDH in severe COVID-19 cases [16]. Our study reinforces the notion that LDH and inflammatory markers are crucial prognostic indicators in COVID-19 patients, particularly those with chronic hepatitis C developing severe disease. We can speculate here that LDH and ferritin values ​​were higher due to the pro-inflammatory status generated by HCV infection, the difference between the subgroups of patients according to the presence of HCV having statistical significance (p<0.01). Regarding the form of the disease, patients with detectable HCV viremia had more severe forms of COVID-19 and more frequently required the administration of corticosteroid and immunomodulatory treatment.

In an Egyptian study of 125 patients diagnosed with COVID-19 and chronic hepatitis C, the most common symptoms were fever, cough, and dyspnea. Factors influencing mortality were male gender, diabetes mellitus, and liver cirrhosis [17]. Similarly, in our analyzed cohort, fever and cough were the most common, followed by digestive manifestations.

In another Italian study of 618 COVID-19 patients, chronic liver disease, male gender, and malignancies were identified as prognostic factors for mortality [18]. In line with previous studies, our cohort identified advanced age and underlying comorbidities such as diabetes and liver cirrhosis as significant risk factors for severe COVID-19 outcomes. Although the presence of cirrhosis was a factor of unfavorable evolution, we did not find a significant difference in this category of patients between those with detectable HCV viremia versus undetectable viremia.

Older age is an independent risk factor associated with mortality and severe disease. A study of 1193 COVID-19 patients showed that age, chronic HCV infection history, and elevated ferritin levels were associated with a high patient mortality rate [11], a finding that we also encountered in our study.

Limitations of study. The retrospective design inherently introduces risks of selection bias and confounding factors that might affect the results. The single-center nature of the study and the relatively small sample size of 89 patients may limit the generalizability of the findings to broader populations. Our study focused exclusively on patients with chronic HCV infection (who have benefited or not from previous antiviral treatment), which inherently predisposes them to a higher likelihood of hepatic function deterioration compared to the general population. We cannot definitively disentangle the independent effects of pre-existing chronic hepatitis C and the SARS-CoV-2 infection on liver injuries and mortality in co-infected individuals. Future prospective studies with larger cohorts are needed to address this critical question.

## Conclusions

Having chronic hepatitis C (HCV) before contracting COVID-19 may worsen the effects of the SARS-CoV-2 virus, leading to a more severe illness. This appears to be true regardless of other health conditions the patient may have, initial blood test results, or any liver damage caused by COVID-19 itself.

Our findings underscore the urgent need for targeted management strategies specifically tailored to this vulnerable patient population. By implementing such strategies, healthcare professionals can potentially improve outcomes and mitigate the risks associated with COVID-19 infection in individuals with chronic hepatitis C.

## Appendices

Appendix 1 

**Table 5:** Univariate logistic regression to preclinical variables relevance in the patient’s outcome ALT: alanine aminotransferase, AST: aspartate aminotransferase, LDH: lactate dehydrogenase.

Predictor	N	Exitus (N)	OR (95% CI)1	P-value
Leukopenia				
Yes	31	3		
No	58	7	1.28 (0.33 to 6.30)	0.734
Thrombocytopenia				
Yes	30	7		
No	58	3	0.18 (0.04 to 0.70)	0.019
Prothrombin Index < 70 (%)	81	9	0.99 (0.96 to 1.02)	0.368
Ferritin/100	51	8	1.18 (1.05 to 1.37)	0.012
Interleukin 6 (pg/ml)	19	2	1.00 (0.99 to 1.01)	0.166
ALT on admission (U/L)	65	4	1.03 (0.95 to 1.13)	0.435
ALT during hospitalization (U/L)	39	2	1.03 (0.93 to 1.14)	0.598
AST on admission (U/L)	4	1	1.13 (0.78 to 2.25)	0.605
AST during hospitalization (U/L)	24	0	1.00 (0.00 to infinite)	0.999
LDH / 100	77	8	2.45 (1.44 to 5.09)	0.006
CT findings				
Yes	17	5	-	
No	72	5	0.18 (0.04 to 0.73)	0.015
Cirrhosis				
Yes	10	3	-	
No	79	7	0.23 (0.05 to 1.23)	0.062

Appendix 2

**Table 6:** COVID-19 in patients with liver cirrhosis depending on HC viremic status RF: risk factor, LDH - lactate dehydrogenase, ALT - alanine aminotransferase.

	Cirrhosis(with detectable viremia) N=10	Without cirrhosis (with detectable viremia) N=26	p
Age (mean)	69.2 (62.2-76-19)	66.9 (61.76-72.17)	0.623
Gender			0.310
Male	4(40.0%)	6(23.1%)	
Female	6(60.0%)	20(76.9%)	
Clinical form of COVID-19			0.895
Moderate/Mild (yes, %)	1(10%)	3(11.5%)	
Severe (yes, %)	9(90%)	23(88.5%)	
RF score (mean)	1.20 (0.32-2.08)	0.96 (0.65-1.27)	0.489
Number of hospitalization days (mean)	14.69 (11.9-17.4)	20.60 (8.6-32.5)	0.136
Pulmonary changes			0.188
No change or interstitial accentuation	0(0.0%)	4(15.4%)	
Infiltrates or gound-glass	10(100%)	22(84.6%)	
Laboratory findings			
LDH (U/L) (mean)	371.1 (291.5-472.2)	381.92 (280.6-461.6)	0.899
ALT (U/L) (mean)	53.8 (6.24-101)	35.7 (28.9-42.5)	0.180
Platelets (mean) (valx10^9^)	159.6 (15.5-303.7)	229 (187-271)	0.178
Ferritin (ng/ml) (mean)	917 (418-1416)	952 (609-1294)	0.902
Leukopenia(yes, %)	6(26.1%)	6(60.0%)	0.063
Treatment			
Corticosteroid(yes)	9(90.0%)	23(88.5%)	0.895
Immunomodulators(yes)	3(30%)	14(53.8%)	0.199
Outcome (n, %)			0.486
Favorable	7(25.0%)	21(75.0%)	
Unfavorably (deceased)	3(37.5%)	5(62.5%)	
